# Concurrent surgical management of pineoblastoma and Chiari type 1.5 malformation: a case report

**DOI:** 10.1186/s12893-025-03403-9

**Published:** 2025-12-06

**Authors:** Hüseyin Yiğit, Abdulkerim Gökoğlu

**Affiliations:** 1 Department of Medical Services and Techniques, Cappadocia University, Nevsehir, Türkiye; 2 Department of Neurosurgery, Konya State Educating & Teaching Hospital, Konya, Türkiye

**Keywords:** Chiari malformation, Hydrocephalus, Neurosurgery, Pineal region tumors, Pineoblastoma

## Abstract

**Purpose:**

Pineoblastoma, a highly aggressive embryonal tumor predominantly affecting the pediatric population, poses significant therapeutic challenges. Current treatment modalities typically involve a combination of surgical resection, chemotherapy, and radiotherapy. While the clinical presentation of pineoblastoma is well-documented, its association with other neurological conditions, particularly Chiari malformations, remains exceedingly rare.

**Case report:**

This report presents a unique case of a 17-year-old male patient diagnosed with both pineoblastoma and Chiari type 1.5 malformation. The patient presented with headache as the sole complaint. Neurological examination revealed nystagmus, quadriparesis, restricted ocular motility in the right eye, and cerebellar signs including a positive Romberg test, dystonia, and dysmetria. Notably, the patient had no history of radiation exposure or known genetic predisposition.

**Conclusion:**

This case marks the initial reported occurrence of concurrently managing a Chiari malformation while surgically removing a pineal region tumor to create a wider and safer surgical corridor. Our combined approach allowed safe and effective tumor resection while optimizing surgical exposure. It may serve as a useful reference for surgeons facing similar complex pathologies.

## Introduction

Pineal region tumors are rare neoplasms of the central nervous system, accounting for less than 1% of diagnosed brain tumors [1]. They are more common in children, representing 3–11% of intracranial masses in pediatric patients [2]. Pineoblastoma is the most aggressive type of pineal tumor, usually occurring in boys in the second decade of life. Hydrocephalus is a common complication, affecting around 90% of patients and causing symptoms like headache and vomiting [3]. Treatment involves removing the tumor mass and addressing any obstructed cerebrospinal fluid pathways. Management is challenging due to the deep-seated location and surrounding neurovascular structures [2]. After surgery, patients typically have a life expectancy of 20–30 months with additional chemotherapy or radiation therapy. Prognostic factors affecting survival include age, co-morbidities, extent of resection, and radiation therapy. New treatment strategies being explored include high-dose chemotherapy, stereotactic radiosurgery, and autologous stem cell therapy with histone deacetylase inhibitors [4].

Pineoblastomas are classified as Grade IV tumors by the World Health Organization, indicating their highly malignant nature and aggressive growth pattern [5]. This case is unique because it involves the simultaneous presentation of pineoblastoma with a Chiari malformation. Chiari malformations (CMs) are a group of anomalies that affect the posterior fossa and/or the craniovertebral junction. They are characterized by the downward herniation of the cerebellar tonsils through the foramen magnum, often with varying degrees of brainstem descent. CMs are classified into four main types (I-IV), with Chiari I malformation being the most common in adults. Chiari type 1.5 malformation is a tonsillar herniation specific to Chiari I malformation, where the brain stem and fourth ventricle are also included in the herniation [6, 7]. This case is the first reported instance of simultaneous surgical management for pineoblastoma and Chiari type 1.5 malformation.

## Case report

We present a 17-year-old male patient with recurrent headaches and progressive neurological symptoms. The timeline of the patient’s medical procedures is summarized in Table 1. Written consent for publication was obtained from the patient’s family. Apart from headaches, the patient had no known medical history, genetic disease, or history of prolonged radiation therapy. During the initial neurological evaluation, limitations in inward gaze of the right eye, right-to-left nystagmus, and tremor in the right hand were noted. Motor examination revealed quadriparesis, loss of deep sensation, hyperactive deep tendon reflexes, and positive Babinski sign in all four extremities. Cerebellar examination showed positive Romberg sign, dysdiadochokinesia, and dysmetria. In May 2020, the patient underwent ventriculoperitoneal (V/P) shunt surgery for acute hydrocephalus at another center. During this procedure, Chiari malformation was diagnosed, and a lesion adjacent to the vermis of the cerebellum was observed. Follow-up without additional surgical intervention was recommended.

**Table 1:** Timeline of patient Management, including diagnosis and surgical intervention

Phase	Date/Time frame	Clinical/Radiological Event	Management/Intervention
Initial Presentation	May 2020	Acute Hydrocephalus (VP shunt placed)	VP Shunt Placement
Recurrence/Worsening	August 2020	Worsening headache, weakness, imbalance, diplopia, diarrhea	Cranial/Cervical MRI and CT scans
Definitive Surgery	September 2020	Pineoblastoma and Chiari 1.5 Malformation	Concurrent Suboccipital Craniectomy/C1 Laminectomy and Pineoblastoma Resection (Single Session)
Postoperative Follow-up	October 2020	Findings Suggestive of Leptomeningeal Metastasis in Spinal Cord	Radiotherapy (IMRT) and Two Courses of Chemotherapy
Long-Term Outcome	5 Year	No Signs of Recurrence or Leptomeningeal Involvement	Uneventful

He presented to our clinic with a headache lasting for about a month, increasing weakness in the upper and lower extremities, walking difficulties, double vision (diplopia), and diarrhea complaints. Cranial and cervical magnetic resonance imaging (MRI) as well as cranial computed tomography (CT) examinations were performed.

### Preoperative radiological findings

**Fig. 1:**
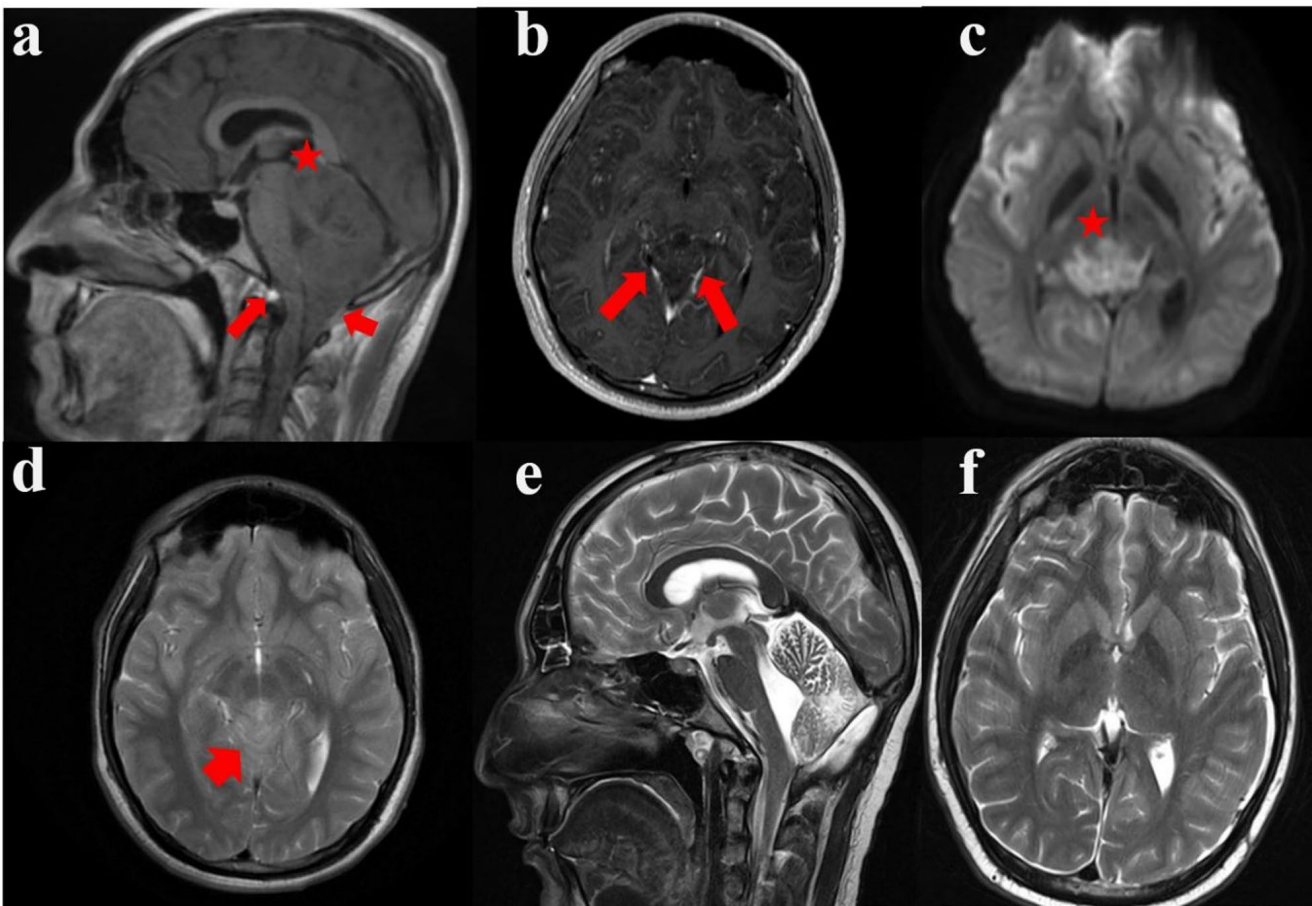
Preoperative and postoperative radiological findings. **a** T1W sagittal MRI reveals cerebellar tonsillar herniation along with caudal herniation of particular portions of the brainstem (often obex of the medulla oblangata) through the foramen magnum; Chiari type 1.5 malformation. **b** T1W axial gadolinium contrast enhanced MR imaging reveals 3 cm in diameter weakly heterogeneous enhancing mass in the pineal gland area compressing deep brain veins. **c** DWI axial MRI reveals diffusion rectricted areas mass lesion, originating from the pineal area extending to the mesencephalon **d** T2W axial MRI reveals hyperintense mass lesion, originating from the pineal area, infiltrating the superior cerebellar peduncles, extending to the mesencephalon and aqueductal region, and narrowing the aqueduct **e** T2W sagittal MRI decompressing of deep cerebral veins due to total resection of tumoral lesion and craniocervical junction due to bilateral cerebellary tonsillectomy and craniectomy **f** T2W axial MRI reveals decompressing of deep cerebral veins due to total resection of tumoral lesion. Red asterix indicates a tumour mass

Initial cranial MRI revealed cerebellar tonsillar herniation, with the cerebellar tonsils extending 20 mm caudal to the medulla through the foramen magnum, consistent with Chiari malformation type 1.5 (basion-opisthion line or McRae line) (Fig. 1a). The odontoid process exhibited an angulation of approximately 75 degrees. Additionally, a 3 cm, weakly contrasted, heterogeneous, T2 hyperintense mass was observed in the pineal region (Fig. 1b). Histopathological analysis of the intraoperative specimen confirmed the diagnosis of pineoblastoma.

Imaging also demonstrated a shunt catheter within the right frontal lateral ventricle. The left lateral ventricle was asymmetrically dilated, while the third ventricle appeared normal. The Evans index was measured at 0.31.

Cranial CT imaging confirmed the presence of the shunt catheter in the right frontal lateral ventricle and the asymmetric dilation of the left lateral ventricle. Obliteration of the cerebellar folia and compression of the fourth ventricle were also noted. A hyperdense, space-occupying lesion, approximately 2 cm in diameter, was visualized within the pineal gland (Fig. 1c).

One month postoperatively, MRI revealed findings suggestive of leptomeningeal metastasis at multiple levels of the spinal cord: C2, C3, C4, T1, T5, T6, T9, T10, and T11. Additional metastatic deposits were observed at the left L3 nerve root and within the dural sac at the L5-S1 level.

### Surgical technique

**Fig. 2:**
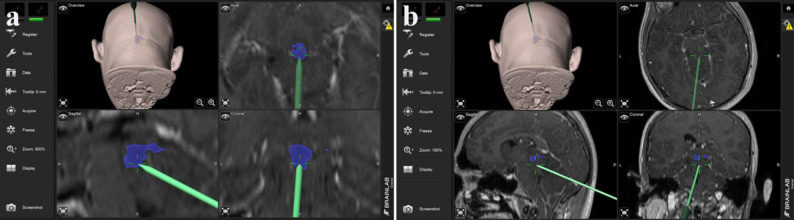
Surgical planning with neuronavigation. **a** Localisation of the pineoblastoma mass. **b** Delineation of the area covered by the pineoblastoma

Under general anesthesia, the patient was positioned in the sitting position with the head flexed at 15 degrees in a Mayfield skull clamp for surgery. Suboccipital craniectomy and C-1 laminectomy were performed with frameless neuronavigation guidance. The entire procedure was conducted under continuous intraoperative neurophysiological monitoring, which included motor evoked potentials and somatosensory evoked potentials to ensure the functional integrity of the brainstem and spinal cord during decompression and tumor resection. The craniectomy area was created to visualize the confluence of sinuses and bilateral transverse sinuses up to two cm laterally in a 5 × 5 cm size. This provided adequate size for both tumor resection and occipitocervical decompression required for Chiari malformation. The pineal region and mass were reached using a supracerebellar infratentorial approach, neuronavigated microscope, and endoscope. A frozen sample was taken from the mass, revealing a diagnosis of pineocytoma. The entire mass was successfully excised, followed by duroplasty and closure (Fig. 2). Two weeks after the operation, the patient started walking without any support.

The decision to perform suboccipital craniectomy and C1 laminectomy concurrently with pineoblastoma resection was based on the need to optimize the operative corridor. The Chiari decompression procedure, typically performed to relieve cervicomedullary compression, facilitated the caudal displacement of the cerebellar hemispheres and tonsils. This decompression provided a wider and safer supracerebellar infratentorial window for total tumor excision, which is technically challenging due to the deep-seated location of the pineal region.

### Pathology

**Fig. 3:**
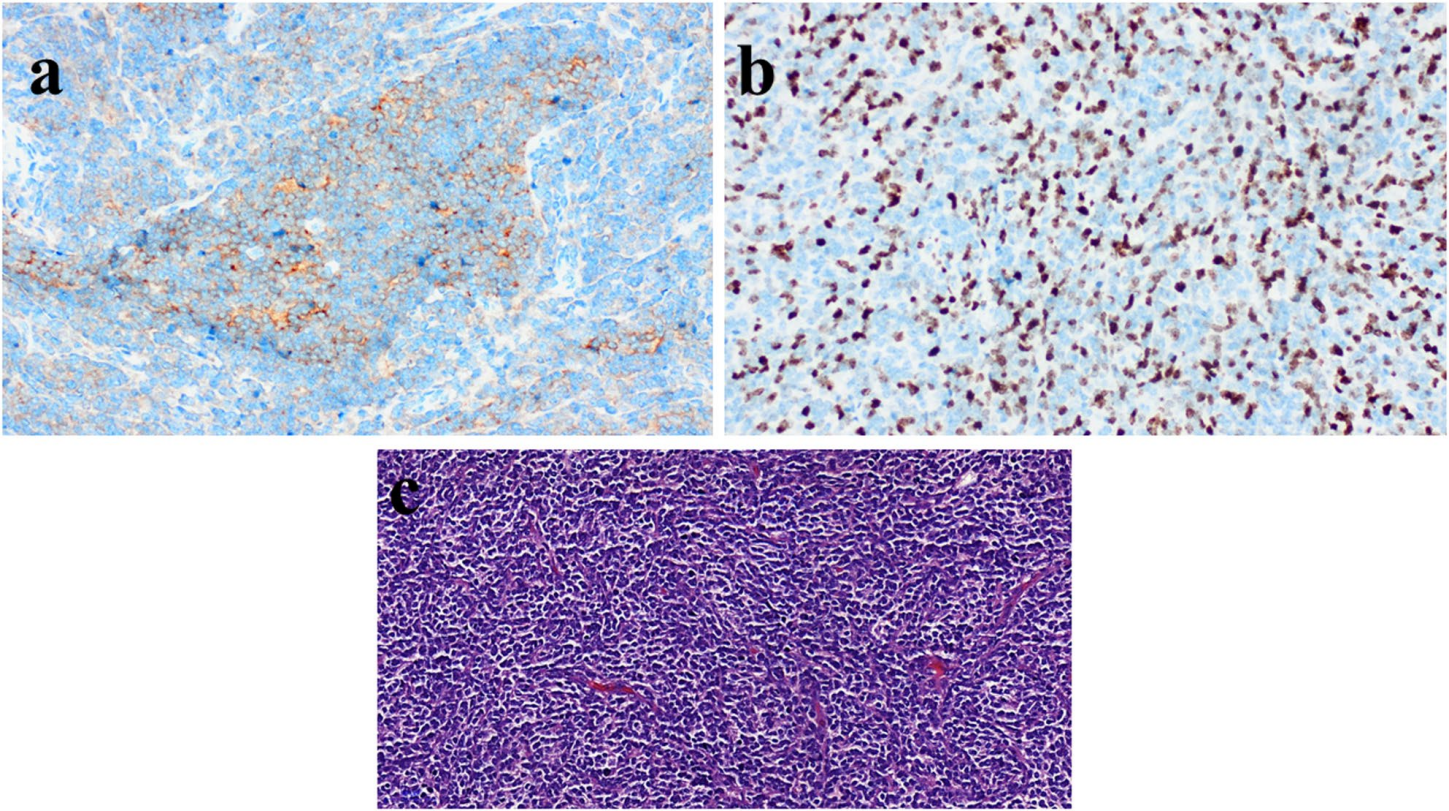
Pathology of pineal blastoma in our case (**a**) The neoplastic cells are synaptophysin positive (Synaptophysin, 10X) (**b**) The mitotic index is high and Ki67 labeling index is 30% (Ki67 20X) (**c**) The tumor is very hypercellular and composed of small blue cells (H&E, 20X)

The macroscopic appearance of the tumor was dirty yellow, soft, and irregular in texture. Microscopic findings indicated a small round blue cell tumor with high mitotic activity, high cellularity, and no necrosis. Immunohistochemical analysis showed negative results for glial fibrillary acid protein (GFAP), cluster of differentiation 20 (CD20), sal-like protein 4 (SALL4), neurofilament protein (NFP), tumor suppressor protein (p53), positive for reticulin fiber (RETICULIN), Synaptophysin, and a Ki-67 of 30%. The tumor was identified as a pineoblastoma (Fig. 3).

### Radiotherapy/chemotherapy

Conformal radiotherapy (IMRT) was administered with doses of 3600 cGy/20 fractions to the cranial area, 4500 cGy/25 fractions to the spinal areas (cervical and thoracic), and 5400 cGy/30 fractions to the primary tumour area. The patient then received two courses of Cyclophosphamide + Etoposide + Carboplatin (5AUC) chemotherapy, followed by Neupogen treatment.

### Postoperative radiological findings

A cranial MRI performed 1 month after the operation revealed a 5 cm cavity extending paramedian to both cerebellar hemispheres at the inferior level of the vermis of the cerebellum. The MRI showed an infiltrative appearance extending to both cerebellar parenchyma and vermis, with markedly increased uptake and soft tissue increase around the cavity. These findings were interpreted as recurrent tumor infiltration in both cerebellar hemispheres and vermis, along with diffuse leptomeningeal involvement around the post-op cavity. Following radiotherapy and chemotherapy, no signs of recurrence or leptomeningeal involvement were observed, indicating that pineoblastoma is responsive to radiation therapy. Throughout the 5-year follow-up of the patient, no abnormalities were detected in any neurological functions (Table 1).

## Discussion

Chiari type 1.5 malformation is a rare condition that is not commonly seen in medical literatüre [7, 8]. Pineoblastoma has never been reported in combination with Chiari malformation, especially Chiari type 1.5 malformation. The patient underwent surgical treatment for Chiari malformation followed by resection of the pineoblastoma. The novelty of our approach lies in the conceptual use of Chiari decompression to actively enlarge the operative field. While the classical supracerebellar infratentorial approach requires significant retraction of the cerebellum, the preceding Chiari decompression effectively mobilized the tonsils and brainstem caudally, providing a more voluminous working corridor and reducing the need for cerebellar retraction. This significantly enhanced the safety profile for achieving total resection of the deep-seated pineoblastoma.

The relationship between hydrocephalus and Chiari malformation is not fully understood. It has been suggested that hydrocephalus may be a cause or consequence of Chiari type 1 malformation [9]. This patient had previously been treated for hydrocephalus at another center, which may have led to Chiari type 1.5 malformation.

**Fig. 4:**
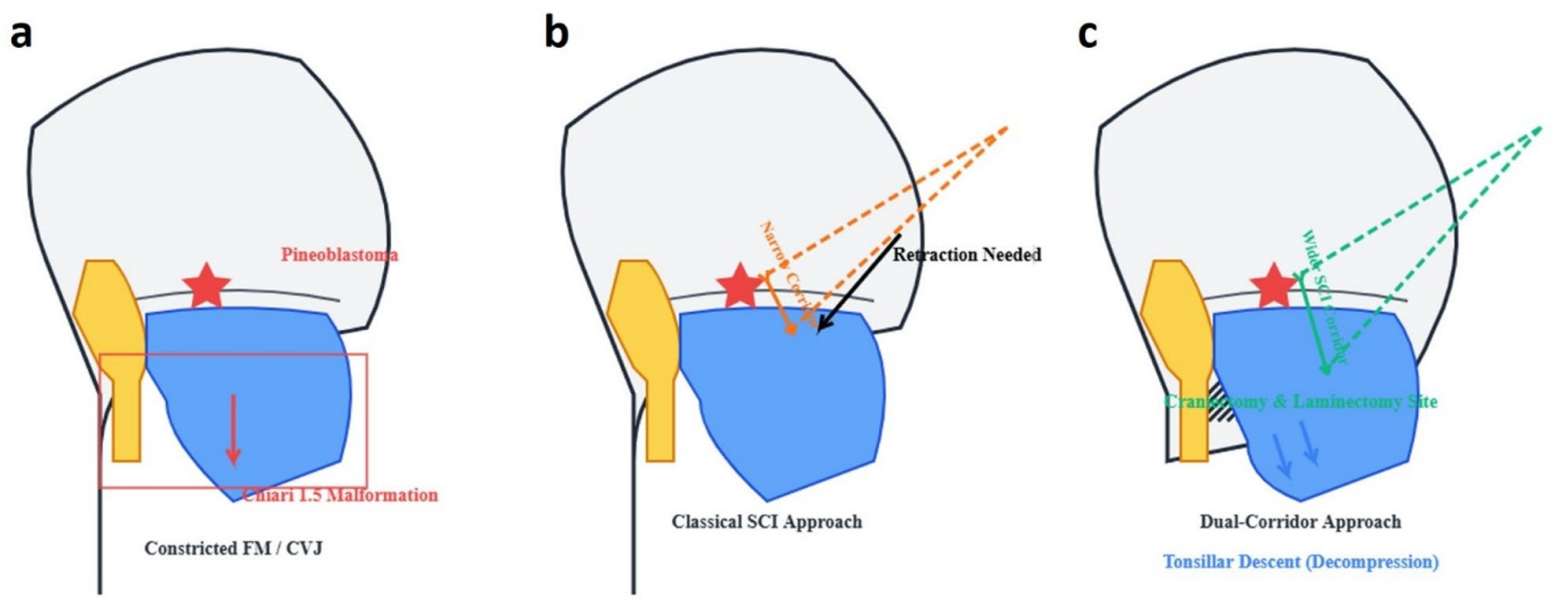
Schematic illustration of the combined pineoblastoma resection and Chiari malformation surgery. **a** Sagittal view showing the pineoblastoma (red star) and the caudal herniation of the cerebellar tonsils (red arrow) through the foramen magnum (FM), constricting the surgical approach. **b** Simulation of the standard supracerebellar infratentorial (SCI) approach. The tonsillar herniation significantly narrows the surgical window (shorter bidirectional arrow), mandating significant cerebellar retraction (black arrow). **c** Our strategy: suboccipital craniectomy/C1 laminectomy (green hatching) facilitates the caudal descent of the tonsils (blue arrows). This active decompression widens the SCI operative corridor (longer bidirectional arrow), optimizing exposure for safe and effective tumor resection. The yellow area represents the brain stem, and the blue area represents the cerebellum. CVJ: craniovertebral joint

Reaching tumors in the deeply seated pineal region presents significant challenges due to the anatomical location of this area [10]. The occipital transtentorial approach carries the risk of causing visual field losses because it requires stretching of the occipital lobe [11], while the posterior interhemispheric approach may compromise critical deep venous structures such as the Galen vein system [12]. Furthermore, both techniques follow a supratentorial course and provide indirect access to the pineal region. Although the telovelar approach is suitable for lesions directed toward the fourth ventricle [13], it does not constitute an appropriate surgical pathway for the removal of pineal tumors. In contrast, the supracerebellar infratentorial approach used in our case preserves visual function by protecting the occipital lobe and provides direct, anatomically appropriate access to the pineal region while minimizing the risk of damage to deep cerebral venous structures due to its infratentorial localization. Our most significant advantage is that we actively widened the supracerebellar infratentorial surgical corridor by simultaneously performing Chiari decompression and moving the cerebellar hemispheres and tonsils caudally (Fig. 4). The wider working space thus created has greatly reduced the need for cerebellar retraction, which is often required in the classic supracerebellar infratentorial approach, and has enabled the safe and complete resection of pineoblastoma. The sitting position preferred in our case not only reduced the amount of bleeding but also provided optimal surgical visibility and access by utilizing gravity without the need for retractors.

## Data Availability

All data relevant to this case report are presented within the manuscript. For any further inquiries, please contact the corresponding author.
